# An Updated Perspective of the Clinical Features and Parathyroidectomy Impact in Primary Hyperparathyroidism Amid Multiple Endocrine Neoplasia Type 1 (MEN1): Focus on Bone Health

**DOI:** 10.3390/jcm14093113

**Published:** 2025-04-30

**Authors:** Ana-Maria Gheorghe, Mihaela Stanciu, Ioana Codruta Lebada, Claudiu Nistor, Mara Carsote

**Affiliations:** 1 PhD Doctoral School, “Carol Davila” University of Medicine and Pharmacy, 020021 Bucharest, Romania; ana-maria.gheorghe@drd.umfcd.ro; 2 Department of Endocrinology, Faculty of Medicine, “Lucian Blaga” University of Sibiu, 550024 Sibiu, Romania; codruta.lebada@ulbsibiu.ro; 3 Department of Endocrinology, Clinical County Emergency Hospital, 550245 Sibiu, Romania; 4 Department 4-Cardio-Thoracic Pathology, Thoracic Surgery II Discipline, “Carol Davila” University of Medicine and Pharmacy, 050474 Bucharest, Romania; 5 Thoracic Surgery Department, “Dr. Carol Davila” Central Military University Emergency Hospital, 010242 Bucharest, Romania; 6 Department of Endocrinology, “Carol Davila” University of Medicine and Pharmacy, 020021 Bucharest, Romania; carsote_m@hotmail.com; 7 Department of Clinical Endocrinology V, “C.I. Parhon” National Institute of Endocrinology, 011863 Bucharest, Romania

**Keywords:** parathyroid, parathyroidectomy, multiple endocrine neoplasia, pituitary, pancreas, neuroendocrine, hormone, osteoporosis, TBS, gene, DXA, fracture, MEN1

## Abstract

**Background**: Multiple endocrine neoplasia type 1 (MEN1)-related primary hyperparathyroidism (MPHPT) belongs to genetic PHPT that accounts for 10% of all PHPT cases, being considered the most frequent hereditary PHPT (less than 5% of all PHPT). **Objective**: We aimed to provide an updated clinical perspective with a double purpose: to highlight the clinical features in MPHPT, particularly, the bone health assessment, as well as the parathyroidectomy (PTx) impact. **Methods**: A comprehensive review of the latest 5-year, English-published, PubMed-accessed original studies. **Results**: The sample-based analysis (n = 17 studies) enrolled 2426 subjects (1720 with MPHPT). The study design was retrospective, except for one prospective and one case–control study. The maximum number of patients per study was of 517. Female predominance (an overall female-to-male ratio of 1.139) was confirmed (except for three studies). Age at MPHPT diagnosis (mean/median per study): 28.7 to 43.1 years; age at PTx: 32 to 43.5 years. Asymptomatic PHPT was reported in 38.3% to 67% of MPHPT. Mean total calcium varied between 1.31 and 2.88 mmol/L and highest PTH was of 317.2 pg/mL. Two studies reported similar PTH and calcaemic levels in MPHPT vs. sporadic PHPT, while another found higher values in MPHPT. Symptomatic vs. asymptomatic patients with MPHPT had similar PTH and serum calcium levels (n = 1). Osteoporosis (n = 8, N = 723 with MPHPT) was reported in 10% to 55.5% of cases, osteopenia in 5.88% to 43.9% (per study); overall fracture rate was 10% (of note, one study showed 0%). Lower bone mineral density (BMD) at DXA (n = 4) in MPHPT vs. sporadic PHPT/controls was found by some studies (n = 3, and only a single study provided third distal radius DXA-BMD assessment), but not all (n = 1). Post-PTx DXA (n = 3, N = 190 with MPHPT) showed a BMD increase (e.g., +8.5% for lumbar spine, +2.1% for total hip, +4.3% for femoral neck BMD); however, post-operatory, BMD remains lower than controls. Trabecular bone score (TBS) analysis (n = 2, N = 142 with MPHPT vs. 397 with sporadic PHPT) showed a higher prevalence of reduced TBS (n = 1) or similar (n = 1). PTx analysis in MPHPT (n = 14): rate of subtotal PTx of 39% to 66.7% (per study) or less than subtotal PTx of 46.9% (n = 1). Post-PTx complications: persistent PHPT (5.6% to 25%), recurrent PHPT (16.87% to 30%, with the highest re-operation rate of 71% in one cohort); hypoparathyroidism (12.4% to 41.7%). Genetic analysis pointed out a higher risk of post-PTx recurrence in exon 10 *MEN1* pathogenic variant. Post-PTx histological exam showed a multi-glandular disease in 40% to 52.1% of MPHPT, and a parathyroid carcinoma prevalence of 1%. **Conclusions**: MPHPT remains a challenging ailment amid a multi-layered genetic syndrome. Current data showed a lower age at MPHPT diagnosis and surgery than found in general population, and a rate of female predominance that is lower than seen in sporadic PHPT cases, but higher than known, for instance, in MEN2. The bone involvement showed heterogeneous results, more consistent for a lower BMD, but not necessarily for a lower TBS vs. controls. PTx involves a rather high rate of recurrence, persistence and redo surgery. About one out of ten patients with MPHPT might have a prevalent fracture and PTx improves the overall bone health, but seems not to restore it to the general population level, despite the young age of the subjects. This suggests that non-parathyroid components and potentially menin protein displays negative bone effects in MEN1.

## 1. Introduction

Multiple endocrine neoplasia type 1 (MEN1) is an autosomal dominant syndrome caused by pathogenic variants of the *MEN1* gene, leading to the development of tumours of various endocrine glands, typically pituitary, pancreas, and parathyroid glands [1,2,3]. The gene is located on chromosome 11q13 and encodes menin, a protein involved in the regulation of transcription and cell division, as well as DNA repair, acting as a tumour suppressor. Loss-of-function variants, such as nonsense or frameshift types, cause aberrant cellular proliferation and tumorigenesis [4,5,6].

While mortality in MEN1 is mainly due to gastro-entero-pancreatic neuroendocrine tumours (GEP-NETs), primary hyperparathyroidism (PHPT) contributes to morbidity through its complications, including osteoporosis and fragility fractures, urolithiasis and chronic kidney disease, and the more recently described cardio-metabolic complications. Moreover, PHPT is a contributor to a lower quality of life due to the physical and neuropsychological symptoms, including depression and anxiety, and negative effects of acute/chronic hypercalcemia [7,8,9].

Genetic forms of PHPT include MEN1, MEN2 (*RET* gene), MEN4 (*CDKN1B/*p27 gene), and hyperparathyroidism-jaw tumour syndrome (*CDC73* gene) [7,8,10]. They account for about 10% of all PHPT cases and are often associated with multi-glandular parathyroid disease and early onset of parathyroid hormone (PTH) excess [9,11]. Even though less than 5% of all cases might suffer from MEN1, MEN1-related PHPT (MPHPT) is considered the most frequent syndromic form [12,13,14]. Due to an increased disease burden, early diagnosis and treatment are essential. In individuals with *MEN1* pathogenic variants, annual calcium and parathyroid hormone (PTH) screening are recommended [13,15,16,17].

Furthermore, MPHPT associates with bone loss/impaired bone strength, leading to osteoporosis and osteoporotic fractures early in the course of disease, frequently more severe compared with sporadic PHPT, noting that acromegaly, secondary diabetes, Cushing’s syndrome, premature hypogonadism, and even some types of GEP-NETs might contribute to additional negative effects on bone health [18,19,20,21,22,23]. Bone involvement (e.g., osteoporosis, fractures, etc.) and other criteria such as young age and non-bone target organ ailments (e.g., kidney) are indications for surgical management of PHPT. Noting the typical multi-glandular form in MPHPT, subtotal parathyroidectomy (STPTx) and total parathyroidectomy (TPTx) are often chosen as surgical approach and redo surgery might be required in many cases [20,21,24].

### Objective

We aimed to provide an updated clinical perspective with a double purpose: highlighting the clinical features in PHPT, particularly, the bone health assessment, as well as the parathyroidectomy impact in individuals confirmed with MEN1.

## 2. Methods

**Figure 1:**
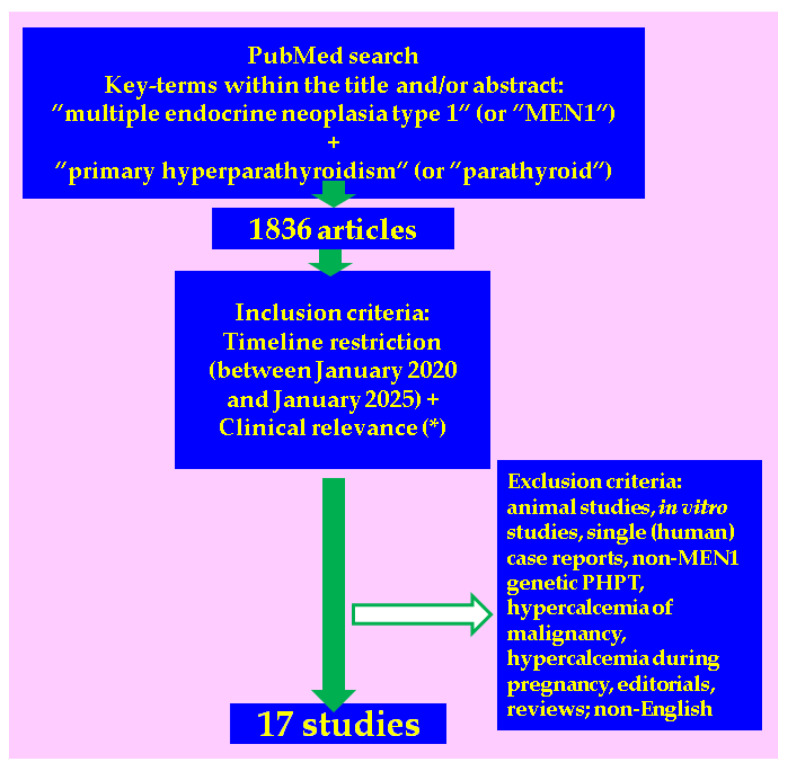
Flowchart of search according to the mentioned methods [25,26,27,28,29,30,31,32,33,34,35,36,37,38,39,40,41] (* clinical presentation in primary hyperparathyroidism, especially bone involvement (e.g., osteoporosis, osteopenia, osteoporotic fractures) and the impact of parathyroidectomy).

This was a PubMed-based comprehensive review that analysed original studies published in English between January 2020 and January 2025. The search was performed using the following keywords in different combinations: “multiple endocrine neoplasia type 1” (or “MEN1”) in combination with “primary hyperparathyroidism” (or “parathyroid”). The studies with focus on patients with MPHPT confirmation were chosen based on their clinical relevance with respect to the clinical presentation of MPHPT, especially bone involvement (osteoporosis, osteopenia, osteoporotic fractures) and the overall impact of surgery in PHPT [25,26,27,28,29,30,31,32,33,34,35,36,37,38,39,40,41]. We looked for the studies that started with a study population diagnosed with PHPT amid MEN1 confirmation compared to other types of PHPT or in relationship with cross-sectional/longitudinal various clinical features within a MPHPT cohort. Exclusion criteria: animal studies, in vitro studies, single (human) case reports, non-MEN1 genetic PHPT, hypercalcemia of malignancy, hypercalcemia during pregnancy, editorials, reviews (Figure 1).

## 3. Results

### 3.1. Studies-Based Analysis

We analysed seventeen original studies published during the last 5 years according to our methods. Out of 2426, 1720 subjects had a confirmation of MPHPT (female-to-male ratio of 916:804; 53.25% females) [25,26,27,28,29,30,31,32,33,34,35,36,37,38,39,40,41] (Table 1).

### 3.2. Main Clinical Features and Mineral Metabolism Findings in MEN1-Related Primary Hyperparathyroidism

The largest study investigated PTx in MEN1 patients and included 517 individuals [27]. The clinical features, particularly the symptoms at diagnosis, were analysed across eleven studies [25,28,29,31,32,34,35,38,39,40,41], while specific mineral metabolism assays were not provided in two studies [27,33]. Asymptomatic PHPT was reported in 38.3% [40] to 67% [39] of all MPHPT cases. The retrospective analysis conducted by Figueiredo et al. [29] showed that most patients with MPHPT were diagnosed due to the associated clinical manifestations (41.2%), 35.3% through MEN1 screening, and 23.5% amid routine blood (biochemistry) analysis [29]. The most frequent manifestation in MPHPT was nephrolithiasis, with prevalence of up to 72% [39]. However, most studies pinpointed a rate between 47.1% [29] and 64.7% [34], while the lowest prevalence (of 27.27%) was identified in a small-sized retrospective study [38]. PHPT-related kidney stones had a similar prevalence in MPHPT and sporadic PHPT (54.5% vs. 62.2%, *p* = 0.594) [25], and did not differ based on the gene pathogenic variant (*p* > 0.05) [28] according to other two studies [25,28]. Hypercalcemia-associated gastrointestinal complains were reported in 10% [39] to 25.8% [31] of the individuals confirmed with MPHPT.

Bone involvement was reported in up to 49.3% [40] of the cases; for instance, bone pain was identified in 17% [39] and 19.3% [40] of MPHPT individuals; while osteoporosis/osteopenia rate varied between 17.6% [29] and 27.27% [38]. Moreover, type 2 diabetes mellitus was found to affect 35% of the MPHPT patients in one cohort (arterial hypertension affected 29% of the enrolled population, while co-occurrence of a thyroid ailment was reported in 20.5% of cases) [34].

Mineral metabolism assays [25,26,28,29,30,31,32,34,35,36,37,38,39,40,41] highlighted mean total calcium between 1.31 mmol/L [26] and 2.88 mmol/L [30] and maximum mean PTH value of 317.2 pg/mL [31]. Two studies reported no statistically significant difference between MPHPT and sporadic PHPT in terms of PTH values (*p* = 0.08 [31], *p* > 0.05 [35]) as well as serum calcium levels (*p* = 0.18 [31], *p* > 0.05 [35]). However, Wang et al. [36] found higher PTH levels in MPHPT vs. sporadic PHPT (470.67 ± 490.74 vs. 217.77 ± 165.60 pg/mL, *p* = 0.001) [36]. Symptomatic and asymptomatic patients with MPHPT had similar PTH (*p* = 0.13) and serum calcium levels (*p* = 0.44) according to another cohort [32] (Table 2).

#### Sub-Analysis of the Bone Health Assessment

Eight studies [25,28,31,34,35,38,40,41] analysed the prevalence of osteoporosis or osteopenia associated or not with (osteoporotic or low-trauma) fragility fractures (a total of 723 patients with MPHPT) [25,28,31,34,35,38,40,41]. The osteoporosis prevalence varied between 10% [28] and 55.5% [41]; osteopenia affected 5.88% [34] to 43.9% [35] of the MPHPT cases and prevalent fractures were detected in almost 10% of all individuals diagnosed with MPHPT (e.g., 7.5% [31]; 9.3% [41]). One retrospective cohort (N = 68) did not identify any prevalent fracture [34]. The analysis in MPHPT vs. sporadic PHPT showed heterogeneous results: Eremkina et al. [25] reported a higher prevalence of a Z-score lower than −2 SD or low-energy fractures in MPHPT vs. sporadic PHPT (59.1% vs. 27%, *p* = 0.026), but a similar rate of the overall fragility fractures (*p* = 0.624) [25]; Wang et al. [41] pinpointed the same rate of osteoporosis in MPHPT vs. sporadic PHPT (54.5% vs. 34%, *p* = 0.302) [41], as did Song et al. [31] in 120 MPHPT subjects vs. 360 cases with sporadic PHPT [e.g., osteoporosis prevalence: 14% vs. 8.2%, *p* = 0.33, prevalence of low bone mineral density (BMD) 46.5% vs. 39.5%, *p* = 0.44, and prevalence of pathological fractures 7.5% vs. 8.9%, *p* = 0.78)] [31] (Table 3).

Baseline Dual-Energy X-Ray Absorptiometry (DXA) parameters were analysed in four studies [25,31,35,41] (N = 252 MPHPT individuals vs. 217 patients with sporadic PHPT) [25,31,35,41]. Lumbar BMD was lower in MPHPT vs. controls (sporadic PHPT) in two studies: 1.02 (0.93, 1.11) vs. 1.15 (1.07, 1.22) g/cm^2^, *p* = 0.002 [25], and 0.91 ± 0.18 vs. 1.01 ± 0.17, *p* < 0.001 g/cm^2^ [31], and similar across another cohort (*p* > 0.05) [35]. Decreased total hip BMD in MPHPT compared to sporadic PHPT was found in two mentioned cohorts: BMD of 0.89 (0.72, 0.92) vs. 0.97 (0.89, 1.10) g/cm^2^, *p* = 0.002, Z-score of −1.00 (−1.80, −0.40) vs. −0.40 (−0.9, 0.40), *p* = 0.018) [25]; respectively, BMD of 0.75 ± 0.30 vs. 0.81 ± 0.23, g/cm^2^, *p* = 0.17, T-score of −1.45 ± 1.00 vs. −0.97 ± 1.38, *p* = 0.01, Z-score of −1.31 ± 0.97 vs. −0.58 ± 1.04, *p* < 0.001) [31]. Similarly, femoral neck BMD was lower in MPHPT vs. sporadic type among the same study population: 0.73 ± 0.35 vs. 0.79 ± 0.18 g/cm^2^, *p* = 0.14, T-score of −1.53 ± 1.02 vs. −0.99 ± 1.09, *p* = 0.002, Z-score of −1.15 ± 1.05 vs. −0.43 ± 1.01, *p* < 0.001 [31], and BMD of 0.81 (0.67, 0.94) vs. 0.94 (0.88, 1.04) g/cm^2^, *p* = 0.001, Z-score of −1.60 (−1.90, −0.80) vs. −0.40 (−1.0, 0.00), *p* = 0.004 [25]. Wang et al. [41] found a statistically significant difference only in total hip and femoral neck Z-score between MEN1 and sporadic PHPT [41]. In contrast with most findings, Marini et al. [35] identified similar DXA results at total hip and femoral neck between MPHPT and sporadic PHPT [35]. A single study provided (N = 59 patients with PHPT, including 22 with MPHPT and 37 with sporadic PHPT) analysed BMD at the third distal radius site, and also reported a lower BMD [0.74 (0.68, 0.85) vs. 0.82 (0.78, 0.89) g/cm^2^; *p* = 0.036] and lower Z-score [−1.50 (−2.3, −0.9) vs. −0.60 (−1.10, 0.00); *p* = 0.007] [25] in MEN1 vs. sporadic cases (Table 4).

Postoperative DXA assessment was provided in three studies (N = 190 subjects with MPHPT [25,26,35]) and overall supported a bone health post-PTx improvement (except for one cohort [35]): e.g., 8.5% increase in lumbar BMD (*p* = 0.008), 2.1% increase in total hip BMD (*p* = 0.005), and 4.3% increase in femoral neck BMD (*p* = 0.007) [25]. Despite post-surgery BMD increase, BMD remains lower than found in the general population; for instance, Kuusela et al. [26] reported that BMD was lower in MPHPT individuals who underwent surgery compared with age- and sex-matched controls at lumbar spine (0.986 ± 0.123 vs. 1.172 ± 0.139 g/cm^2^, *p* < 0.001), total hip (0.931 ± 0.130 vs. 1.022 ± 0.128 g/cm^2^, *p* = 0.004), and femoral neck (0.782 ± 0.119 vs. 0.967 ± 0.129 g/cm^2^, *p* < 0.001) [26] (Table 5).

Two retrospective studies [25,31] reported trabecular bone score (TBS) values in 142 patients diagnosed with MPHPT vs. 397 cases with sporadic PHPT [25,31]. The largest of them (N = 120 subjects with MPHPT vs. 360 age- and sex-matched individuals with sporadic PHPT) identified a higher prevalence of a TBS value lower of equal to 1.230 (53.4% vs. 26.7%, *p* < 0.001) in MPHPT vs. sporadic type [31], suggesting a negative effect of PHPT on bone microarchitecture in MEN1. Moreover, TBS increased with serum ionized calcium in MPHPT (B = 0.275, SE = 0.132, *p* = 0.04) [31]. The other cohort [25] highlighted a similar TBS in MPHPT vs. sporadic PHPT [1.39 (1.32–1.45) vs. 1.49 (1.40–1.51), *p* = 0.136], but decreased scores amid 3D DXA evaluation, with post-PTx improvement [25] (Table 6).

### 3.3. Parathyroidectomy Outcome in MEN1

Fourteen studies [26,27,28,29,30,31,32,33,34,35,37,38,39,40] analysed PTx-related impact in MPHPT [N = 1644 patients with MPHPT, median/mean age at PTx varied between 30 (22, 38) years [28] and 43.4 ± 14.1 years [37]). Seven studies [27,28,29,32,33,34,35] reported STPTx as the most common surgical approach, with a prevalence between 39% [35] and 66.7% [32]. Kuusel et al. [26], however, found that most patients (46.9%) underwent less than subtotal PTx (<STPTx) [26]. In contrast, Choi et al. [37] found that over half of the patients (51.51%) underwent total PTx (TPTx) [37], and, similarly, Manoharan et al. [39] reported TPTx as the most frequent surgical procedure (42.7% of the subjects) [39].

The most common post-operatory complications were: persistent hyperparathyroidism, recurrent hyperparathyroidism and hypoparathyroidism. Persistent values of high PTH were reported in up to 25% [29] of the subjects (with a minimum rate of 5.6% [39]). Recurrence occurred in about a third of the cases according to the majority of the mentioned studies [31,34,39] (lowest rate of 16.7% [29], if any at all [38]). Libansky et al. [30] analysed data regarding reoperation in MPHPT and found a high recurrence rate of 71.4% and a persistence of disease in 28.6% of cases [30]. The risk of recurrence was higher in patients with exon 10 pathogenic variant [OR (95% CI) = 2.19 (1.31–3.69), *p* = 0.003] and in patients who initially underwent <STPTx [OR (95% CI) = 2.61 (2.03–3.31), *p* < 0.001] [27]. Post-PTx hypoparathyroidism occurred in 12.4% [35] up to 41.7% [29] of patients with MPHPT who underwent PTx. Patients with 4-gland resection (or more than 4-gland) had a lower chance of recovery from iatrogenic hypoparathyroidism [OR (95% CI) = 0.19 (0.05–0.72), *p* = 0.02] [33]. Permanent laryngeal nerve palsy did not occur in most studies [28,34,38,39], except for one cohort (a rate of 3.7%) [30].

Some studies compared the surgical approaches: Santucci et al. [27] found in a large retrospective cohort on 517 patients with MPHPT who underwent PTx that <STPTx was associated with a higher recurrence (68.5% vs. 45%, *p* < 0.001), a higher persistence rate (18% vs. 3.2%, *p* < 0.001), and a lower rate of hypoparathyroidism at six months (3.4% vs. 22.7%, *p* < 0.001) and at one year (2.3% vs. 19.5%, *p* < 0.001) compared with STPTx [27]. Similarly, Shariq et al. [28] reported a lower persistence/recurrence in patients who underwent STPTx or TPTx compared with <STPTx (61% vs. 60% vs. 84%, *p* = 0.0003) [28]. A lower recurrence rate in STPTx (10.1% vs. 21.3%, *p* = 0.03) and TPTx (4.4% vs. 21.3%, *p* = 0.001) vs. <STPTx was also reported by Manoharan et al. [39], but with a similar persistence rate across study sub-groups (*p* = 0.052) [39]. A retrospective study conducted by Choi et al. [37] analysed parathyroid venous sampling and found no statistically significant difference in terms of PHPT recurrence (*p* = 1.00). However, the sub-group of patients who underwent parathyroid venous sampling had a lower rate of permanent hypoparathyroidism (0% vs. 50%, *p* = 0.033) [37] (Table 7).

#### 3.3.1. Pre-Operatory Imaging Evaluation of the Parathyroid Masses in MEN1

Amid this study-based analysis [24,25,26,27,28,29,30,31,32,33,34,35,36,37,38,39,40,41], we identified two studies to address the issue of pre-surgery localization of the parathyroid masses [36,38]. Wang et al. [36] compared the detection rate in MPHPT vs. sporadic PHPT and reported similar rates of an adequate imaging diagnosis (87% vs. 93.9%, *p* = 0.33). In terms of ultrasound features, individuals with MPHPT had more often round lesions (80% vs. 25.8%, *p* < 0.001), while other characteristics such as irregular shape of the parathyroid mass, vague boundary, heterogeneity, and abundant blood flow were similar between the study sub-groups [36]. Gauthé et al. [38] compared different preoperative localization techniques and pinpointed the highest sensitivity for the combination of three imaging techniques, ultrasonography and methoxyisobutylisonitrile labelled with technetium-99 m (sestaMIBI) and fluorine-18 positron emission tomography associated with computed tomography (FCH-PET/CT) (90%). In terms of specificity, however, FCH-PET/CT reached 92%, followed by ultrasonography with a specificity of 91%. The highest predictive value was of 91% for ultrasonography or FCH-PET/CT. Negative predictive value was highest in ultrasonography and sestaMIBI combined with FCH-PET/CT. Overall, while ultrasonography had an elevated specificity and positive predictive value, its accuracy was limited by a low sensitivity of maximum 60%. The best accuracy was confirmed for a combination between ultrasonography and either FCH-PET/CT and/or sestaMIBI [38] (Table 8).

#### 3.3.2. Post-Parathyroidectomy Pathological Exam: Parathyroid Masses in MEN1

Five studies, including 345 patients with MPHPT, provided a post-operatory histological analysis [26,31,36,38,40]. Multi-glandular disease was reported in 40% [36] to 52.1% [31], with a higher prevalence in MPHPT vs. sporadic PHPT (52.1% vs. 10%, *p* < 0.001 [31], respectively, 40% vs. 10%, *p* = 0.003 [36]). Atypical parathyroid neoplasms occurred in 1.8% [40] of the cases, while parathyroid carcinoma associated a prevalence of around 1% [31,40]. Three studies [26,38,40] used previous terms of “hyperplasia” (for multi-glandular disease) and “adenoma” (for parathyroid tumours) [7,42,43,44], and two of them reported hyperplasia in most patients (59.4% [26], and 69% [38]), while the third one identified an adenomas prevalence of 57.1% [40] (Table 9).

#### 3.3.3. Management of the Primary Hyperparathyroidism and Its Impact on the Quality of Life in MEN1 Subjects

We identified a single study regarding the quality of life in MPHPT according to our methods (this was a prospective analysis on 30 surgery candidates). Both the physical component and the mental component were similar at baseline and six and twelve months postoperatively. However, when symptomatic and asymptomatic patients were compared, symptomatic patients had lower physical and mental health scores, corresponding to worse quality of life, compared to their asymptomatic counterparts. The study also reported a small negative correlation between total parathyroid volume and the role-functioning physical score [r = −0.44, CI = (−0.70, −0.09), *p* = 0.01], suggesting that larger parathyroid masses are associated with a lower quality of life. Small positive correlations between the remnant parathyroid volume and the physical component summary score (r = 0.3625, *p* = 0.049), and mental component summary score (r = 0.3807, *p* = 0.038) were also identified [32] (Table 10).

## 4. Discussion

### 4.1. MEN1: A Complex Lens to Look at Primary Hyperparathyroidism

In this large analysis, a heterogeneous panel of clinical features, including in the bone field and surgery-related aspects have been identified [25,26,27,28,29,30,31,32,33,34,35,36,37,38,39,40,41]. To summarize, the sample-based clinical perspective (n = 17 studies) enrolled 2426 subjects and 1720 of them had MPHPT. A female predominance in terms of female-to-male ratio of 1.139 was identified [25,26,27,28,29,30,31,32,33,34,35,36,37,38,39,40,41]. The study design was mostly retrospective, with two exceptions: one prospective [32] and one case–control [41] study. The publication timeline showed four studies published in 2024, three in 2023, three in 2022, two in 2021, and five articles in 2020 [25,26,27,28,29,30,31,32,33,34,35,36,37,38,39,40,41]. The number of patients per study varied: <100 persons [25,26,29,32,34,36,37,38,39], between 101 and 209 individuals [28,30,35,40,41], and up to 480 [31] and 517 people [27].

Generally, PHPT represents a central feature of MEN1, being the initial and most frequent manifestation in most MEN1 cases, with a prevalence of 90% up to 100% [39,45,46,47]. Moreover, MEN1 is the most common cause of familial PHPT and typically its onset is within the second decade of life, but it may develop even before the age of five years [47,48,49]. Considering that early detection is the key factor for an optimum management and for providing a prompt intervention, as similarly found in other poly-glandular endocrine ailments, current guidelines recommend that individuals with a positive family history for MEN1 should undergo *MEN* genetic testing [19,50]. In subjects coming from MEN1 families, or those positive at *MEN1* genetic screening, regular biochemical screening helps identifying the underlying hormonal anomalies early in the course of the syndrome. MPHPT screening may be initiated even during childhood under certain circumstances [49,51]. However, extensive testing amid serial screening and invasive medical interventions might impact the long-term quality of life [52]. The definitive cure of MPHPT is PTx, as seen in sporadic cases. Even though symptomatic disease typically appears after the third decade of life, biochemical screening may identify the condition in the asymptomatic or normocalcemic stages. A consensus regarding the timing of PTx in patients with mild, asymptomatic disease, however, has not been reached yet [53,54]. Regarding the optimum surgical approach, some authors suggested that STPTx balances the risk of postoperative complications such as permanent hypoparathyroidism vs. recurrent/persistent PHPT [55,56,57].

Of important note, in this analysis [25,26,27,28,29,30,31,32,33,34,35,36,37,38,39,40,41] we only included patients who were confirmed with MPHPT amid various sub-groups of assessments, and not individuals who were *MEN1*-positive plus MPHPT-negative under surveillance protocols. The baseline study population was stratified as follows:

- single MPHPT cohort [34,38,40]
- patients with MPHPT vs. sporadic PHPT [25,31,33,35,38,41]
- subjects with MPHPT vs. (PHPT-free) controls [26]
- surgery candidates who were confirmed with MPHPT [27,32,37,39] or MPHPT vs. non-MPHPT [30,36]
- MEN1 patients (including patients who presented MPHPT) [28]
- individuals with familial type of PHPT (including MPHPT) [29]

The female prevalence in MPHPT sub-groups (rate per study) reached maximum values of 65% [35], 70% [41], 76% [30], and 81% [25]; most studies reported a prevalence of approximately 50–58% as follows: 50% [29,39], 51% [26,33], 53 [32], 55% [27], 56% [28], and 58% [31,40]. Three studies showed a more frequent male population: 74% [36], 63% [38], and 58% [34]. None of these cohorts highlighted the traditional female prevalence of 80–90%, as found in sporadic PHPT, while, for instance, in MEN2 usually women and men are equally affected [1,2,14].

Age-analysis per study [mean ± SD or median (IQR)] indicated:

- Age at MPHPT diagnosis (years): 28.7 ± 13.6 [26]; 30 (22, 38) [28]; 34 (21, 69) [38]; 34.1 ± 13.5 [36]; 35.2 ± 14 [34]; 36 (28, 39) [25]; 38.64 ± 15.25 [41]; 43 ± 15.5 y [40]; and 43.1 ± 14.2 [29]
- Age at *MEN1* genetic testing (years): 30.3 ± 16.3 [26]
- Age at MEN1 diagnosis (years): 39 ± 13.06 [34]; and 35 (18, 76) [38]
- Age at PTx (years): 32 ± 12.7 [33]; 36.2 (25, 48) [27]; 38 (22, 44) [32]; 38.7 ± 2.46 [30]; 43.4 ± 14.1 [37]; 35 (18–70) [39]; 43.5 (31.5, 52) [31]

Generally, the *MEN1* gene, located on chromosome 11q13, encodes a tumour suppressor protein (menin) and most pathogenic variants in MEN1 are frameshift variants (42% of cases), followed by missense variants (25% of cases) [58,59]. Menin plays important roles in cell proliferation by regulating the gene transcription, and in stability of the genome [60,61,62], and it also induces epigenetic changes which favour tumour proliferation by regulating non-coding RNAs and interacting with chromatin-associated protein complexes [63,64]. In the parathyroid glands, an additional mechanism involves the tumour growth factor (TGF) beta/Smad signalling pathway. In order to inhibit cell proliferation in the parathyroid glands, TGF-beta requires activation by menin, therefore loss-of-function variants of menin promote parathyroid cell proliferation [65]. In order for tumorigenesis to occur, usually, there is a loss of heterozygosity generated by a second unidentified event [45]. The importance of menin in parathyroid proliferation is also highlighted by the presence of *MEN1* somatic variants, such as intragenic deletions, in sporadic parathyroid tumours [66]. As mentioned, the evidence regarding genotype–phenotype correlation between MEN1 pathogenic variants and the clinical picture is scarce [67]. Some explanations include epigenetic factors, such as the pathway of miR-24 microRNAs, which silences menin in the parathyroid [68]. Moreover, recent data suggest that other genetic features such as *Kras* variants may influence the phenotypic outcome of *MEN1* pathogenic variants [69].

According to the current study-based analysis, Shariq et al. [28] reported that in patients who developed MPHPT-truncating variants in exons 2, 9, or 10 were more frequent compared to patients who did not develop MPHPT (38% vs. 13%, *p* = 0.05). Moreover, these variants were also associated with a younger age at disease onset (27 years vs. 31 years, *p* = 0.007). Most patients who underwent TPTx had truncating variants of *MEN1* exons 2, 9, or 10 (80%), while 60% of STPTx and 66% of <STPTx were performed in other *MEN1* variants. In terms of the clinical manifestation, the study did not find any statistically significant difference between patients with variants in exons 2, 9, or 10 and other variants [28]. Notably, Santucci et al. [27] found that pathogenic variants in exon 10 increased the risk of recurrent PHPT following STPTx (OR = 2.19 (1.32, 3.69), *p* = 0.003). Variants in exon 2 and 9 did not increase the risk of recurrence (*p* = 0.767, and *p* = 0.111 respectively) [27]. Recently, a potential connection between pathogenic variants in exon 2, 9, or 10 and the patients’ age at MPHPT onset, the post-surgical outcome and the risk of recurrence has been pinpointed [27,28].

Apart from MPHPT, MEN1 includes various endocrine tumours/NETs such as GEP-NETs, pituitary NETs (PitNETs), thymus and bronchial carcinoids, and adrenocortical tumours [70,71,72,73,74]. GEP-NETs occur in 30% to 80% of MEN1 patients [75,76,77]. The histological type varies e.g., gastrinomas, insulinomas, glucagonomas, as well as non-functioning NETs [77,78,79]. Early screening, even in teenagers, has been shown to be beneficial, while genetic testing might represent a prognostic tool and may guide the management considering that pathogenic variants in exon 2, 9, and 10 were shown to associate an increased risk of metastatic disease, in spite of no clear genotype–phenotype correlations as found, for instance, in MEN2 [80,81,82]. MEN1-related PitNETs may be hormonally active, as well, such as lactotroph, somatotroph and corticotroph PitNEts, or non-functional NETs; MEN1-PitNETs might have similar manifestations to sporadic PitNETs and generally they impact the overall mortality and morbidity in MEN1 in addition to GEP-NETs [83,84]. Notably, multiple complications and comorbidities due to an excessive hormonal activity such as insulin resistance, diabetes, and carcinoid syndrome associated or not with carcinoid heart disease might dominate the clinical picture and increase the syndrome burden [51,85,86,87]. Other less specific clinical elements include MEN1-related cutaneous tumours such as lipomas, angiofibromas, and collagenomas that have been found with a higher rate than general population [88,89,90] and uncommon neoplasia like leiomyomas and breast and ovarian tumours, as well as T-cell lymphoma [91,92,93].

### 4.2. Contributing Factors for Bone Loss in MEN1: From Parathyroidectomy Timing and Benefits to the Impact of Non-Parathyroid Components

One of the main complications of PHPT is the skeletal involvement, which includes fragility fractures, osteoporosis/osteopenia in addition to other clinical elements such as kidney stones, renal function impairment, cardio-metabolic issues, and acute complications of hypercalcemia [15,94,95,96]. Typically, PHPT leads to the resorption of cortical bone, particularly at the distal radius [97]. However, in patients with PHPT, fracture risk is elevated for all osteoporotic sites, including vertebral [98,99]. This risk does not seem to be directly dependent on PHPT severity, nor DXA-BMD [100]. Moreover, even normocalcemic variant has an increased prevalence of fractures and osteoporosis similar to hypercalcaemic PHPT (as found for other complications, including, renal, etc.) [101,102,103,104]. Modern techniques such as lumbar DXA-based TBS and high-resolution peripheral quantitative computed tomography (HR-pQCT) have shown that bone microarchitecture is also disturbed in PHPT; a fact that might explain the gap between BMD values and the co-presence of an increased risk of fragility fractures [105,106,107]. Assessing the bone status in PHPT (regardless of an underlying genetic syndrome) represents the key aspect for an adequate management, considering the elevated burden of fractures, hence, the importance of establishing a prompt indication for PTx that might help restoring the bone health (to some extent) [14,106].

**Figure 2:**
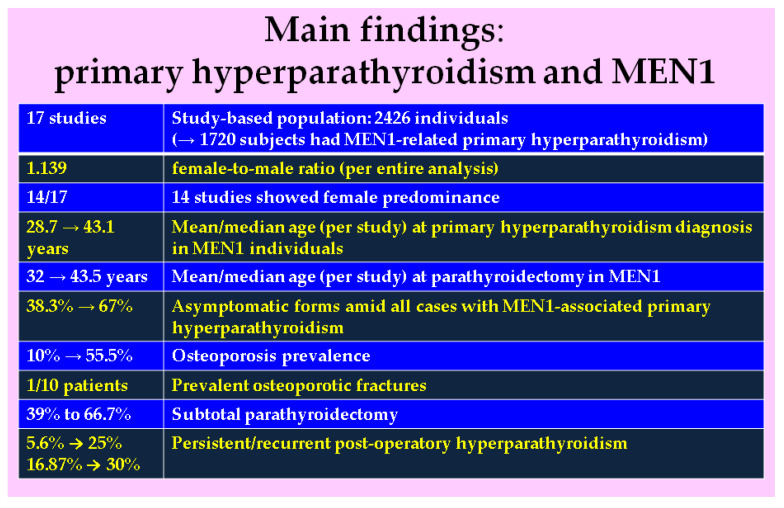
Main findings in sample-focused analysis [25,26,27,28,29,30,31,32,33,34,35,36,37,38,39,40,41].

In this analysis [25,26,27,28,29,30,31,32,33,34,35,36,37,38,39,40,41], asymptomatic PHPT was reported in 38.3% to 67% of MPHPT. Mean total calcium varied between 1.31 and 2.88 mmol/L while highest PTH was of 317.2 pg/mL. Two studies reported similar PTH and calcaemic levels in MPHPT vs. sporadic PHPT, and another found higher values in MPHPT. Symptomatic vs. asymptomatic patients with MPHPT had similar PTH and serum calcium levels (n = 1). Osteoporosis (n = 8, N = 723 with MPHPT) was reported in 10% to 55.5% of cases, osteopenia in 5.88% to 43.9% (per study). Overall, the fractures rate was of 10% (of note, one study showed 0%). Lower BMD at central DXA (n = 4) in MPHPT vs. sporadic PHPT/controls was found by some studies (n = 3, and only a single study provided third distal radius DXA-BMD assessment), but not all (n = 1). Post-PTx DXA (n = 3, N = 190) showed a BMD increase (maximum of +8.5% for lumbar spine); however, post-operatory, BMD remains lower than (non-PHPT) controls. TBS analysis, despite offering a lower level of statistical significance at this point (n = 2, N = 142 with MPHPT), showed a higher prevalence of reduced TBS (n = 1) or similar (n = 1) [25,28,31,34,35,38,40,41] (Figure 2).

Globally, MPHPT plays a central role in the impairment of bone health in MEN1. Prior data [108] and recent data (as we could identify them, [25,31]) have showed a lower BMD and/or a higher prevalence of osteoporosis compared to PHPT in the general population, suggesting that further mechanisms are at play in bone metabolism disturbances. However, this aspect of BMD damage was not supported by all studies over the time assessed [35,41,109]. Generally, the bone involvement in MEN1 originates from multifactorial, intricate pathways, extending beyond the effects of MPHPT toward the co-diagnosis of PitNETs and GEP-NETs and their complications (e.g., persistent, endogenous hypercortisolism, diabetes or early hypogonadism, etc.) or to potential negative effects of menin itself on the bone metabolism [110,111,112]. Somatotroph, lactotroph, and corticotroph PitNETs contribute to a high bone resorption level and consecutive increased fracture risk [113]. In somatotroph PitNETs, prolonged exposure to high levels of growth hormone supplementary impairs the bone microarchitecture and increases the vertebral fractures risk [113,114,115]. Lactotroph PitNETs-related hyperprolactinemia is the main contributing factor to bone loss and osteoporosis via central hypogonadism [116,117]. Moreover, prolactin has a direct impact on skeleton through an inhibitory effect on osteoblasts [118] and enhances the bone turnover, especially resorption [119]. Corticotroph PitNETs also disturb the bone metabolism, particularly causing vertebral fractures in a third to a half of the patients, depending on the disease severity [120,121]. Central hypogonadism, as a frequent complication of PitNETs, develops either due to tumour compression, the inhibitory effect of prolactin on gonadotropin releasing hormone (in lactotroph PitNETs), or as a complication of pituitary surgery or radiation [122,123]. Of note, hypogonadism may lead to osteoporosis in both men and women [124]. Oestrogen is involved in bone formation by activating Wnt/β-catenin signalling and inhibits resorption by increasing osteoprogerin expression and inhibiting the action of nuclear factor-κβ ligand [125], while testosterone, on the other hand, contributes both directly by binding to androgen receptors in osteoblasts, osteoclasts and osteocytes, and indirectly through its conversion to oestrogen by aromatase [125,126]. GEP-NETs may also impair bone health either through the secretion of bone active molecules such as gut serotonin, or PTH-related peptide, through the presence of bone metastases or due to malnutrition/malabsorption or general altered status [127,128]. Co-occurrence of vitamin D deficiency might contribute to the risk of fall and impair the mineralization [129].

The function of menin in bone cells is incompletely understood so far. In vitro and animal studies have shown menin’s involvement in the bone microenvironment by regulating the formation, differentiation and function of the osteoblasts, as well as the osteocyte-osteoclast interaction [130,131,132]. A more recent murine model has shown an increased osteoclastogenesis with preserved osteoblasts number which caused a lower bone density as assessed by DXA and altered trabecular bone structure as evaluated by 3-D micro CT in *MEN1* knockout mice [133]. Moreover, recent in vitro studies have pointed out the role of menin in the matrix mineralization [134].

From a larger view, as part of a familial cancer syndrome, MPHPT impacts the quality of life in addition to the issues related to the oncological component, including metastases, aggressiveness of the associated tumours/NETs and multimodal management, anxiety related to the disease, serial testing, familial implications of harbouring the pathogenic variants, etc. [135,136,137,138]. The surgical burden, not only of PTx, and the high risk of post-operatory persistent or recurrent disease contributes to a reduced quality of life in MPHPT [139,140]. Surgical complications, such as permanent hypoparathyroidism with life-long requirements of calcium and vitamin D supplementation, increase the burden due to potential frequent episodes of hypocalcaemia and financial load [141]. However, in spite of the particularities and multiple contributing factors, there are no specific questionnaires for assessing quality of life in MEN1 patients, making the estimation imprecise [59]. We mentioned a single small-sized study with respect to assessing the quality of life in MEN1 and PHPT with no clear conclusion [32]. The issue is not only the need of a larger study population, and longitudinal data, but also the necessity of testing different scales in order to provide an adequate evaluation for this particular ailment.

To summarize, the PTx analysis in MPHPT (n = 14) showed a rate of STPTx of 39% to 66.7% (per study) or <STPTx of 46.9% (n = 1). Post-PTx complications involved persistent PHPT (5.6% to 25%), recurrent PHPT (16.87% to 30%, with highest re-operation rate of 71% in one cohort), and hypoparathyroidism (12.4% to 41.7%). Genetic analysis pointed out a higher risk of post-PTx recurrence in exon 10 *MEN1* pathogenic variant. Post-PTx histological exam showed a multi-glandular disease in 40% to 52.1% of MPHPT, and a parathyroid carcinoma prevalence of 1% [25,26,27,28,29,30,31,32,33,34,35,36,37,38,39,40,41].

Integrating MPHPT to the panel of genetic PHPT might be challenging despite the progress of modern clinical medicine. In patients with familial PHPT, a negative testing for *MEN1*, potentially involves MEN2, MEN4, familial isolated PHPT, or hyperparathyroidism jaw-tumour syndrome [142,143]. In contrast with MEN1, MEN4, a far less common syndrome, leads to a milder clinical picture, and later onset of PHPT. Genetic testing for MEN4 in MEN1-like patients with negative menin testing might improve the overall management, considering the weak genotype–phenotype correlation [144,145]. Moreover, in MEN2, PHPT is less frequently found, with a prevalence of around 30%, and only exceptionally represents the first manifestation of the syndrome. Most commonly, the first manifestation of MEN2 is medullary thyroid carcinoma, which strongly correlates with the underlying *RET* pathogenic variant (which is not the case in MEN1 for *MEN1*) [146,147,148].

### 4.3. Current Limits and Further Research

As limits of the current sample-based analysis, we mention the narrative review took place amid a single database search. To date, the topic remains complex and dynamic, with many areas still unknown. For instance, which is the particular genetic and epigenetic configuration to be associated with a particular clinical picture, including bone health damage in MPHPT, is still an open issue. With regard to the practical surgical approach, the timing and the type of techniques, as mentioned, still involve some areas of uncertainty, and further studies are necessary. We found no particular data regarding a non-parathyroid-related bone involvement and fracture risk in MEN1 vs. MPHPT-associated bone damage, and further controlled longitudinal studies might highlight the differences. Fracture risk assessment algorithms and practical models of estimation might help the long-term management in MEN1 patients with a higher risk of developing MPHPT or a history of prior (unsuccessful) PTx. Additional experimental studies are useful to point out the role of menin in the parathyroid and bone cells under physiological and pathological circumstances of the syndrome. On the practical side, we should mention the need of using more scales to assess and follow the quality of life in MEN1/MPHPT individuals, including those with osteoporosis and prevalent fragility fractures. The optimum medical and surgical management in normocalcemic variant and mildly symptomatic MEN1 cases (particularly for those who do not share the panel of classical PTx indications) remains an open issue, as well, and currently it is mostly a matter of a tailored decision.

## 5. Conclusions

MPHPT, the central manifestation of MEN1, is an important source of morbidity and syndrome-related burden, impairing not only the overall health, particularly of the bone status, but also the quality of life. While there is still a lack of genotype–phenotype correlation, recent data connected truncating variants in exons 2, 9, and 10 of *MEN1* gene to a younger age at onset and the need of a more extensive surgery in MPHPT. MPHPT remains a challenging ailment amid a multi-layered genetic syndrome. Current data show a lower age at MPHPT diagnosis and surgery than found in the general population, and a rate of female predominance that is lower than that seen in sporadic PHPT cases, but higher than known, for instance, in MEN2. The bone involvement showed heterogeneous results, more consistent for a lower BMD, but not necessarily for a lower TBS vs. controls. PTx involves a rather high rate of recurrence, persistence, and redo surgery. However, a surgical approach needs to take into consideration the balance between the risk of disease persistence/recurrence and the risk of post-PTx hypoparathyroidism. About one out of ten patients with MPHPT might have a prevalent fracture and PTx improves the overall bone health, but seems not to restore it to the general population level, despite the young age of the subjects. This suggests that non-parathyroid components, and potentially menin protein, display negative bone effects in MEN1.

## Figures and Tables

**Table 1:** Overview of original studies regarding MEN1-related primary hyperparathyroidism (of note, the study design and the studied sub-groups were introduced according to the original studies) [25,26,27,28,29,30,31,32,33,34,35,36,37,38,39,40,41].

First Author/Year/Reference	Study Design	Study Population
Eremkina/2024 [25]	Retrospective study	**N = 59 with MHPT vs. sporadic PHPT**; F:M = 6.375:1 (86.44% females) N1 = 22 with MPHPT; F:M = 18:4 (81.81% females); age [median (IQR)] = 36 (28, 39) y N2 (from N1) = 11 with MPHPT with dynamic follow-up N3 = 37 with sporadic PHPT; F:M = 8.25:1 (89.18% females); age [median (IQR)] = 34 (30, 38) y N4 (from N3) = 14 with sporadic PHPT with dynamic follow-up
Kuusela/2024 [26]	Observational study	**N = 70 with MPHPT vs. controls ** N1 = 35 with MPHPT; F:M = 18:17 (51% females); age (mean ± SD) = 42.8 ± 15.7 y Age at genetic testing (mean ± SD) = 30.3 ± 16.3 y Age at MPHPT diagnosis (mean ± SD) = 28.7 ± 13.6 y N2 = 35 age- and sex-matched controls; F:M = 18:17 (51% females); age (mean ± SD) = 43.2 ± 9.71 y
Santucci/2024 [27]	Retrospective cohort study	**N = 517 surgery candidates (who underwent PTx for MPHPT)**; F:M = 287:230 (55.5% females); Age at diagnosis [median (IQR)] = 36.2 (25, 48) y N1 = 178 who underwent <STPTx; F:M = 101:77 (57% females); age at diagnosis [median (IQR)] = 36.4 (25, 49) Y N2 = 339 who underwent STPTx; F:M = 186:153 (55% females); age at diagnosis [median (IQR)] = 36.1 (25, 46) Y
Shariq/2024 [28]	Retrospective study	**N = 209 with MEN1** N1 = 194 with MPHPT; F:M = 109:85 (56.18% females); age [median (IQR)] = 30 (22, 38) y N2 = 73 with MPHPT and truncating variant in exon 2, 9 or 10; F:M = 39:34 (53% females); age [median (IQR)] = 50 (39, 62) N3 = 121 with MPHPT and other pathogenic variants; F:M = 70:51 (58% females); age [median (IQR)] = 55 (40,64)
Figueiredo/2023 [29]	Retrospective analysis	**N = 48 with familial PHPT**; F:M = 24:24 (50% females); age (mean ± SD) = 40 ± 15.5 yN1 = 17 (35.4%) with MPHPT; F:M = 8:9 (47.1% females)Age at PHPT diagnosis (mean ± SD) = 43.1 ± 14.2 yAge at first manifestation (mean ± SD) = 37.7 ± 17.6 y
Libánský/2023 [30]	Retrospective study	**N = 101 surgery candidates (who underwent PTx for PHPT vs. MPHPT)** N1 = 78 with PHPT and reoperation; F:M = 60:18 (76.92% females); age (mean ± SD) = 58.37 ± 1.56 y N2 = 27 with MPHPT; F:M = 17:10 (62.96% females); age (mean ± SD) = 38.7 ± 2.46 y
Song/2023 [31]	Retrospective observational study	**N = 480 with MPHPT vs. sporadic PHPT** N1 = 120 with MPHPT; F:M = 70:50 (58.33% females); age [median (IQR)] = 43.5 (31.5, 52) y N2 = 360 with sporadic PHPT; F:M = 255:105 (70.83% females); age [median (IQR)] = 52 (40.5, 61) y N3 (from N1) = 86 with MPHPT with bone data; F:M = 39:47 (45.34% females); age at onset [median (IQR)] = 44 (31.5, 55) y N4 (from N2) = 86 age and sex matched with sporadic PHPT and bone data; F:M = 32:54 (37% females); age at onset [median (IQR)] = 48.5 (38, 57) y
Bresci/2022 [32]	Prospective study	**N = 30 surgery candidates (who underwent PTx for MPHPT)**; F:M = 16:14 (53.33% females) Age at PTx [median (IQR)] = 38 (22, 44) y
Landry/2022 [33]	Retrospective study	**N = 206 surgery candidates (who underwent PTx for MPHPT)**; F:M = 106:100 (51% females) Age at first PTx (mean ± SD) = 32 ± 12.7 y
Yavropoulou/2022 [34]	Retrospective cohort study	**N = 68 with MPHPT**; F:M = 29:39 (42.6% females) Age at MEN diagnosis (mean ± SD) = 39 ± 13.06 y Age at MPHPT diagnosis (mean ± SD = 35.2 ± 14 y
Marini/2021 [35]	Retrospective study	**N = 180 with MPHPT vs. sporadic PHPT** N1 = 133 with MPHPT; F:M = 87:46 (65.4% females); age at MPHPT diagnosis (mean ± SD) = 34.1 ± 13.5 y N2 = 47 with sporadic PHPT; F:M = 44:3 (93.6% females)
Wang/2021 [36]	Retrospective cohort study	**N = 45 surgery candidates (who underwent PTx for MPHPT vs. sporadic PHPT + thyroidectomy for thyroid nodules**; F:M = 12:33 (26.7% females) N1 = 15 with MPHPT; F:M = 4:11 (26.7% females); age at thyroidectomy (mean ± SD) = 52.87 ± 9.92 y N2 = 30 with sporadic PHPT (age and sex matched with N1); F:M = 8:22 (26.7% females); age at thyroidectomy (mean ± SD) = 53.43 ± 9.2 y
Choi/2020 [37]	Retrospective study	**N = 33 surgery candidates (who underwent PTx for MPHPT)**; age (mean ± SD) = 43.4 ± 14.1 y N1 = 12 with MPHPT who underwent <STPTx; age (mean ± SD) = 37.4 ± 8.9 y N2 = 4 with MPHPT who underwent STPTx; age (mean ± SD) = 42 ± 10.8 y N3 = 17 with MPHPT who underwent TPTx; age (mean ± SD) = 48 ± 16.49 y
Gauthé/2020 [38]	Retrospective study	**N = 22 with MPHPT**; F:M = 6:16 (37.5% females) Age at MEN1 diagnosis [median (IQR)] = 35 (18, 76) y Age at MPHPT diagnosis [median (IQR)] = 34 (21, 69) y
Manoharan/2020 [39]	Retrospective study	**N = 89 surgery candidates (who underwent PTx for MPHPT)**; F:M = 44:45 (49.43% females); age [median (range)] = 35 (18–70) y N1 = 28 with MPHPT who underwent SGE; age [median (range)] = 40 (range 18–67) N2 = 23 with MPHPT who underwent STPTx; age [median (range)] = 36 (range 18–68) N3 = 38 with MPHPT who underwent TPTx; age [median (range)] = 32 (range 18–70)
Song/2020 [40]	Retrospective study	**N = 153 with MPHPT** N1 = 150 with MPHPT without PC/APN; F:M = 87:63 (58% females); age at disease onset (mean ± SD) = 43 ± 15.5 y N2 = 3 with MPHPT and PC/APN; F:M = 2:1; age at disease onset >49 y
Wang/2020 [41]	Case control study	**N = 116 with PHPT (MPHPT sub-group) vs. controls (sporadic PHPT sub-group) ** N1 = 58 with PHPT; F:M = 41:17 (70.68% females); age (mean ± SD) = 49.9 ± 15.1 y N2 = 58 age and sex matched controls; F:M = 41:17 (70.68% females); age (mean ± SD) = 50.6 ± 15.7 y N3 (from N1) = 11 with MPHPT; F:M = 8:3 (72.72% females); age (mean ± SD) = 38.64 ± 15.25 y N4 (from N2) = 47 with sporadic PHPT; F:M = 33:14 (70.21% females); age (mean ± SD) = 52.57 ± 13.99 y N3 vs. N4 age *p* = 0.005

Abbreviations: APN = atypical parathyroid neoplasm; F:M = female-to-male ratio; IQR = interquartile range; MPHPT = MEN1-related PHPT; N = number of patients; PHPT = primary hyperparathyroidism; PC = parathyroid carcinoma; PTx = parathyroidectomy; SD = standard deviation; SGE = single gland excisions; vs. = versus; y = years (**magenta = baseline study population/type of study population that have been addressed according to the original study**).

**Table 2:** Evaluation of mineral metabolism and main clinical features in primary hyperparathyroidism and MEN1 according to original studies [25,26,28,29,30,31,32,34,35,36,37,38,39,40,41].

Reference	Assessment of the Calcium Metabolism	Clinical Features
[25]	N1 vs. N3 PTH: 131.6 (95.92, 198.3) vs. 117.3 (102.3, 169.5) pg/mL, *p* = 0.931 Albumin corrected Ca: 2.69 (2.62, 2.80) vs. 2.69 (2.63, 2.77) mmol/L, *p* = 0.911 24-h urine calcium: 8.22 (6.42, 10.29) vs. 8.61 (6.60, 10.90) mmol/L, *p* = 0.651	Nephrolithiasis: 54.5% (12/22) vs. 62.2% (23/37), *p* = 0.594
[26]	N1: PTH: 96.6 ± 68.9 ng/L N1 vs. N2: Ionized Ca: 1.31 ± 0.12 vs. 1.24 ± 0.03 mmol/L, *p* = 0.001 24-h urinary calcium: 6.7 ± 4.9 vs. 2.3 ± 1.2, *p* < 0.001	NA
[28]	N2 vs. N3: PTH: 72 (59, 110) vs. 83 (60, 114) pg/mL Total Ca: 11 (10.6, 11.3) vs. 11 (10.4, 11.3) mg/dL	Bone mineral density loss: 45% (87/194) Nephrolithiasis: 60% (115/194) N2 vs. N3: Median (IQR) age at PHPT onset: 27 (21, 34) vs. 31 (22, 41) y, *p* = 0.007 Bone mineral density: *p* > 0.05 Nephrolithiasis: *p* > 0.05
[29]	PTH: 169.9 (210.5) pg/mL Total Ca: 11.7 ± 1.2 mg/dL	N1: Diagnosis of PHPT: Screening: 35.3% (6/17) Clinical manifestations: 41.2% (7/17) Routine blood analysis: 23.5% (4/17) Nephrolithiasis: 47.1% (8/17) Osteoporosis/osteopenia: 17.6% (3/17) Chronic kidney disease: 11.8% (2/17)
[30]	PTH: 18.12 ± 3.74 pmol/L Total Ca: 2.88 ± 2.46 mmol/L	NA
[31]	N1 vs. N2 PTH: 317.2 (130.1, 353.0) vs. 514.9 (135.0, 520.2) pg/mL, *p* = 0.08 Total Ca: 2.84 ± 0.24 vs. 2.90 ± 0.34 mmol/L, *p* = 0.18 iCa: 1.40 ± 0.14 vs. 1.45 ± 0.26 mmol/L, *p* = 0.29 24-h urinary Ca: 7.7 (5.5, 10.2) vs. 7.8 (4.7, 10.9) mmol/day, *p* = 0.90	N1 vs. N2 Skeletal symptoms: 10.8% vs. 24.4%, *p* = 0.002 Pathologic fracture: 7.5% vs. 8.9%, *p* = 0.78 Subperiosteal absorption: 1.7% vs. 17.2%, *p* < 0.001 Osteitis fibrosa cystica: 3.3% vs. 10.0%, *p* = 0.04 Osteomalacia: 1.7% vs. 5.6%, *p* = 0.13 Gastrointestinal symptoms: 25.8% vs. 27.2%, *p* = 0.86 Urinary involvement: 54.2% vs. 35.6%, *p* < 0.001 Hypercalcaemic crisis: 0.8% vs. 10.6%, *p* = 0.002 Asymptomatic: 38.3% vs. 39.2%, *p* = 0.96
[32]	Preoperative: Ca: 10.8 (10.4, 11.1) mg/dL 9.4 and PTH: 104 (76, 137) pg/mL Symptomatic vs. asymptomatic: PTH: 111 (78, 171) vs. 101 (58, 116) pg/mL, *p* = 0.13 Total Ca: 10.7 (10.3, 11.1) vs. 10.9 (10.4, 11.2) mg/dL, *p* = 0.44	Symptomatic: 63.3% (19/30) Asymptomatic: 36.7% (11/30)
[34]	NA	PHPT as first manifestation: 64.7% (44/68) Comorbidities: 72% (49/68) Type 2 diabetes mellitus: 35% (24/68) Hypertension: 29% (20/68) Thyroid pathology: 20.5% (14/68) Death: 11.7% (8/68) Osteoporosis: 17.6% (11/68) Osteopenia: 5.88% (4/68) Nephrolithiasis: 64.7% (22/68) Nephrocalcinosis: 1.47 (1/68)
[35]	N1 vs. N2: PTH: 17.2 ± 17.2 vs. 19.0 ± 18.0 pmol/L, *p* > 0.05 Ionized Ca: 5.67 ± 0.43 vs. 5.55 ± 0.35 mg/dL, *p* > 0.05 Total Ca: 10.5 ± 1.5 vs. 10.3 ± 0.9 mg/dL, *p* > 0.05 24-h urinary calcium: 328.7 ± 155.4 vs. 269.5 ± 123.7 mg/24 h, *p* > 0.05	Nephrolithiasis: 47.4% (63/133)
[36]	N1 vs. N2: PTH: 470.67 ± 490.74 vs. 217.77 ± 165.60 pg/mL, *p* = 0.001	NA
[37]	N1 vs. N2 vs. N3: PTH: 108.8 ± 37.7 vs. 138.1 ± 52.2 vs. 190.9 ± 90.6, *p* = 0.017	NA
[38]	PTH: 54.9 (33.9, 114.1) ng/L Albumin corrected Ca: 2.70 (2.51, 3.01) mmol/L Ionized Ca: 1.41 (1.33, 1.60) mmol/L	Osteopenia/osteoporosis: 27.27% (6/22) Nephrolithiasis/nephrocalcinosis: 27.27% (6/22)
[39]	PTH median (range): 106.5 (51–2040) pg/mL Total Ca median (range): 2.85 (2.30–3.70) mmol/L	Symptoms: 33% (29/89) Nephrolithiasis: 72% (21/89) Ulcer: 10% (3/89) Bone pain: 17% (5/89) N1 vs. N2 vs. N3: Symptoms: 50% (14/28) vs. 30.1% (9/23) vs. 100% 31.5% (12/38), *p* > 0.05 Nephrolithiasis: 25% (7/28) vs. 21.7% (5/23) vs. 23.7% (9/38), *p* > 0.05 Ulcer: 3.5% (1/28) vs. 8.7% (2/23) vs. 0% (0/38), *p* > 0.05 Bone pain: 7.1% (2/28) vs. 8.7% (2/23) vs. 2.6% (1/38), *p* > 0.05
[40]	N1: PTH: 185.5 (108.3, 297.0) pg/mL Total Ca: 2.78 (2.61, 2.88) mmol/L 24-h urinary calcium: 7.68 (5.09, 10.28) mmol/day	N1: Gastrointestinal involvement: 21.3% (32/150) Bone involvement: 49.3% (74/150) Bone pain: 19.3% (29/150) Pathological fracture: 9.3% (14/150) Subperiosteal absorption: 8% (12/150) Osteitis fibrosa cystica: 3.3% (5/150) Osteoporosis: 28.6% (43/150) Urinary tract involvement: 46.7% (70/150) N2: Nephrolithiasis: 100% (3/3) Bone pain and osteoporosis 66.6% (2/3) Gastrointestinal symptoms: 33.3% (1/3)
[41]	N3 vs. N4: Total Ca: 2.75 ± 0.13 vs. 2.81 ± 0.31 mmol/L, *p* = 0.526 iCa: 1.42 ± 0.06 vs. 1.41 ± 0.18 mmol/L, *p* = 0.779 Serum PTH: 141.5 (78.7, 245.7) vs. 185.2 (39.9, 1891.5) pg/mL, *p* = 0.207	N3 vs. N4 Asymptomatic: 54.5% (6/11) vs. 38.3% (18/47), *p* = 0.325

Abbreviations: Ca = calcium; iCa = ionized serum calcium; N = number of patients; NA = not available; PTH = parathormone; PHPT = primary hyperparathyroidism; Total Ca = total serum calcium. Studies sub-groups have been introduced in Table 1.

**Table 3:** Osteoporosis/osteopenia and prevalent fractures in MEN1-related primary hyperparathyroidism [25,28,31,34,35,38,40,41].

Reference	Prevalence of Osteoporosis/Osteopenia	Prevalence of Low Energy Fractures
[25]	N1 vs. N3: Z-score < −2.0 SD or low-energy fractures: 59.1% (13/22) vs. 27% (10/37), *p* = 0.026	N1 vs. N3: 9.1% (1/22) vs. 5.4% (2/37), *p* = 0.624
[28]	N2 vs. N3: Osteoporosis: 15% (11/73) vs. 7% (9/121) and osteopenia: 34% (25/73) vs. 35% (42/121)	NA
[31]	N3 vs. N4: 14% vs. 8.2%, *p* = 0.33 BMD below expected for age: 46.5% vs. 39.5%, *p* = 0.44	N1 vs. N2: Pathological fractures 7.5% (9/120) vs. 8.9% (32/360), *p* = 0.78 N3 vs. N4: Pathological fractures 8.1% (7.86) vs. 5.8% (5/86), *p* = 0.76
[34]	Osteoporosis: 17.6% (11/68) and osteopenia: 5.88% (4/68)	Fragility fracture: 0%
[35]	N1:Osteoporosis: 40.9% (27/66) and osteopenia: 43.9% (29/66) N2: Osteoporosis: 66.0% (31/47) and osteopenia: 27.6% (13/47)	NA
[38]	Osteopenia/osteoporosis: 27.27% (6/22)	NA
[40]	N1: Osteoporosis: 28.6% (43/150) N2: Bone pain and osteoporosis 66.6% (2/3)	N1: Pathological fracture: 9.3% (14/150)
[41]	N3 vs. N4: 54.5% (6/11) vs. 34.0% (16/47), *p* = 0.302	NA

Abbreviations: N = number of patients; NA = not available; SD = standard deviation; vs. = versus. Studies sub-groups have been introduced in Table 1.

**Table 4:** Preoperative/baseline DXA assessment in cases with MEN1 and primary hyperparathyroidism [25,31,35,41].

Reference	Lumbar Spine BMD/T-Score Mean ± SD or Median (IQR)	Total Hip BMD/T-Score Mean ± SD or Median (IQR)	Femoral Neck BMD/T-Score Mean ± SD or Median (IQR)
[25]	N1 vs. N3: BMD = 1.02 (0.93, 1.11) vs. 1.15 (1.07, 1.22), *p* = 0.002 g/cm^2^ Z-score = −1.50 (−1.90, −1.00) vs. −0.50 (−1.20, −0.10), *p* = 0.012	N1 vs. N3: BMD = 0.89 (0.72, 0.92) vs. 0.97 (0.89, 1.10) g/cm^2^, *p* = 0.002 Z-score = −1.00 (−1.80, −0.40) vs. −0.40 (−0.9, 0.40), *p* = 0.018	N1 vs. N3: BMD = 0.81 (0.67, 0.94) vs. 0.94 (0.88, 1.04) g/cm^2^, *p* = 0.001 Z-score = −1.60 (−1.90, −0.80) vs. −0.40 (−1.0, 0.00), *p* = 0.004
[31]	N3 vs. N4 BMD = 0.91 ± 0.18 vs. 1.01 ± 0.17, *p* < 0.001 g/cm^2^ T-score = −1.69 ± 1.48 vs. −0.94 ± 1.40, *p* < 0.001 Z-score = −1.40 ± 1.39 vs. −0.50 ± 1.21, *p* < 0.001	N3 vs. N4 BMD = 0.75 ± 0.30 vs. 0.81 ± 0.23, *p* = 0.17 g/cm^2^ T-score = −1.45 ± 1.00 vs. −0.97 ± 1.38, *p* = 0.01 Z-score = −1.31 ± 0.97 vs. −0.58 ± 1.04, *p* < 0.001	N3 vs. N4 BMD = 0.73 ± 0.35 vs. 0.79 ± 0.18, *p* = 0.14 g/cm^2^ T-score = −1.53 ± 1.02 vs. −0.99 ± 1.09, *p* = 0.002 Z-score = −1.15 ± 1.05 vs. −0.43 ± 1.01, *p* < 0.001
[35]	N1 vs. N2 BMD = 0.884 ± 0.154 vs. 0.855 ± 0.133 g/cm^2^, *p* > 0.05 T-score = −1.7 ± 1.4 vs. −2.1 ± 1.2, *p* > 0.05	N1 vs. N2 BMD = 0.843 ± 0.177 vs. 0.816 ± 0.141 g/cm^2^, *p* > 0.05 T-score = −1.3 ± 1.0 vs. −1.5 ± 0.9, *p* > 0.05	N1 vs. N2 BMD = 0.704 ± 0.120 vs. 0.702 ± 0.150 g/cm^2^, *p* > 0.05 T-score = −1.7 ± 0.9 vs. −1.9 ± 1.2, *p* > 0.05
[41]	N3 vs. N4 T-score = −2.0 (−3.0, 1.7) vs. −1.2 (−5.2,0.8), *p* = 0.498 Z-score = −1.8 (−2.5, 1.9) vs. −0.3 (−2.7, 2.3), *p* = 0.042	N3 vs. N4 T-score = −1.6 (−2.9, 1.3) vs. −1.1 (−3.3,0.6), *p* = 0.052 Z-score = −1.6 (−2.8, 1.6) vs. −0.8 (−3.2, 0.9), *p* = 0.042	N3 vs. N4 T-score = −1.8 (−3.1, 0.7) vs. −1.4 (−3.5, 0.7), *p* = 0.218 Z-score = −1.7 (−2.5, 1.6) vs. −0.8 (−3.0, 1.3), *p* = 0.054

Abbreviations: BMD = bone mineral density; IQR = interquartile range; N = number of patients; vs. = versus. Studies sub-groups have been introduced in Table 1.

**Table 5:** DXA assessment after parathyroid surgery for primary hyperparathyroidism in MEN1 subjects [25,26,35].

Reference	Lumbar BMD	Total Hip BMD	Femoral Neck BMD
[25]	+8.5%, *p* = 0.008	+2.1%, *p* = 0.005	+4.3%, *p* = 0.007
[26]	N1 vs. N2 BMD = 0.986 ± 0.123 vs. 1.172 ± 0.139 g/cm^2^, *p* < 0.001 T-score = −0.79 ± 1.14 vs. −0.15 ± 1.19, *p* = 0.03 Z-score = −0.29 ± 1.14 vs. −0.10 ± 1.18, *p* = 0.49	N1 vs. N2 BMD = 0.931 ± 0.130 vs. 1.022 ± 0.128 g/cm^2^, *p* = 0.004 T-score = −0.44 ± 0.98 vs. −0.19 ± 1.01, *p* = 0.309 Z-score = −0.10 ± 0.80 vs. −0.04 ± 0.95, *p* = 0.778	N1 vs. N2 BMD = 0.782 ± 0.119 vs. 0.967 ± 0.129 g/cm^2^, *p* < 0.001 T-score = −0.99 ± 0.89 vs. −0.45 ± 1.03 *p* = 0.012 Z-score = −0.37 ± 0.67 vs. −0.19 ± 0.98 *p* = 0.356
[35]	N1BMD = 0.818 ± 0.157 vs. 0.879 ± 0.164 g/cm^2^, *p* > 0.05 T-score = −2.3 ± 1.3 vs. −1.7 ± 1.4, *p* > 0.05	N1 BMD = 0.801 ± 0.161 vs. 0.841 ± 0.170 g/cm^2^, *p* > 0.05 T-score = −1.6 ± 0.9 vs. −1.2 ± 1.0, *p* > 0.05	N1 BMD = 0.673 ± 0.114 vs. 0.697 ± 0.128 g/cm^2^, *p* > 0.05 T-score = −1.9 ± 0.9 vs. −1.6 ± 1.0, *p* > 0.05

Abbreviations: BMD = bone mineral density; N = number of patients; Studies sub-groups have been introduced in Table 1.

**Table 6:** Trabecular bone score evaluation in patients with MEN1 and primary hyperparathyroidism [25,31].

Reference	Trabecular Bone Score Median (IQR)	3D DXA Analysis Median (IQR)
[25]	N1 vs. N3: 1.39 (1.32–1.45) vs. 1.49 (1.40–1.51), *p* = 0.136	N1 vs. N3: Cortical sBMD TH = 131.15 (106.96–150.63) vs. 151.95 (141.89–163.72) g/cm^2^, *p* = 0.001 Cortical sBMD FN = 102.06 (92.54–118.58) vs. 130.10 (119.68–138.09) g/cm^2^, *p* < 0.001 Trabecular vBMD TH = 142.22 (105.29–181.17) vs. 168.81 (150.22–212.23) g/cm^3^, *p* = 0.029 Trabecular vBMD FN = 181.93 (154.69–235.27) vs. 237.74 (212.92–265.67) g/cm^3^, *p* = 0.008 Cortical vBMD TH = 724.79 (652.67–779.78) vs. 800.74 (751.19–857.710) g/cm^3^, *p* = 0.007 Cortical vBMD FN = 713.81 (671.471–768.502) vs. 797.82 (758.03–858.38) g/cm^3^, *p* = 0.002 Cortical Thickness TH = 1.77 (1.65–1.83) vs. 1.910 (1.86–2.01) mm, *p* < 0.001 Cortical Thickness FN = 1.48 (1.40–1.59) vs. 1.65 (1.49–1.80) mm, *p* = 0.002 N2 before vs. after PTx: Cortical sBMD TH = 135.70 (100.65–153.83) vs. 147.71 (106.21–168.08) g/cm^2^, *p* = 0.001 Cortical sBMD FN = 112.20 (95.04–123.62) vs. 121.33 (101.16–132.55) g/cm^2^, *p* = 0.001 Trabecular vBMD TH = 157.17 (113.95–177.26) vs. 172.62 (120.75–226.64) g/cm^3^, *p* = 0.019 Trabecular vBMD FN = 204.18 (170.26–226.75) vs. 207.64 (170.02–286.12) g/cm^3^, *p* = 0.019 Cortical vBMD TH = 745.44 (597.69–776.26) vs. 761.41 (614.26–835.44) g/cm^3^, *p* = 0.005 Cortical vBMD FN = 738.11 (636.25–781.54) vs. 767.69 (667.84–816.35) g/cm^3^, *p* = 0.019 Cortical Thickness TH = 1.79 (1.68–1.96) vs. 1.878 (1.73–2.01) mm, *p* = 0.005 Cortical Thickness FN = 1.61 (1.50–1.71) vs. 1.65 (1.58–1.71) mm, *p* = 0.007
[31]	N3 vs. N4 1.230 < TBS < 1.310: 20.9% vs. 26.7%, *p* = 0.47 TBS ≤ 1.230: 53.4% vs. 26.7%, *p* < 0.001 Serum ionized calcium and TBS in N3: B = 0.275, SE = 0.132, *p* = 0.04	

Abbreviations: BMD = bone mineral density; DXA = Dual-Energy X-Ray Absorptiometry; FN = femoral neck; IQR = interquartile range; N = number of patients; sBMD = surface bone mineral density; SE = standard error; TBS = trabecular bone score; TH = total hip; vBMD = volumetric bone mineral density; vs. = versus. Studies sub-groups have been introduced in Table 1; red font highlights bone mineral density-based assays amid 3D DXA.

**Table 7:** Analysis of parathyroidectomy performed in MEN1-related primary hyperparathyroidism [26,27,28,29,30,31,32,33,34,35,37,38,39,40].

Reference	Age at PTx	Surgical Approach	Post-Surgery Outcome
[26]	33.3 ± 13.7 y	<STPTx: 46.9% (15/32) STPTx: 15.6% (5/32) TPTx: 37.5% (12/32)	Recurrent PHPT: <STPTx: 86.7% (13/15) STPTx: 0% TPTx: 66.7% (8/12) Persistent PHPT: 62.9% (22/35)
[27]	N: 37.7 (27, 49) y N1: 37.0 (26, 50) y N2: 37.9 (28, 48) y	<STPTx: 34.43% (178/517) STPTx: 65.57% (339/517)	N vs. N1 vs. N2: Recurrent PHPT: 53.2% vs. 68.5% vs. 45%, *p* < 0.001 Persistent PHPT: 8.3% vs. 18% vs. 3.2%, *p* < 0.001 Hypoparathyroidism at 6 mo: 16% vs. 3.4% vs. 22.7%, *p* < 0.001 Hypoparathyroidism at 12 mo: 13.5% vs. 2.3% vs. 19.5%, *p* < 0.001 Risk of recurrence OR (95% CI): Exon 10 pathogenic variant: 2.19 (1.31–3.69), *p* = 0.003 <STPTx: 2.61 (2.03–3.31), *p* < 0.001 Sex: *p* = 0.490 Age at surgery: *p* = 0.612 Exon 2 pathogenic variant: *p* = 0.767 Exon 9 pathogenic variant: *p* = 0.111
[28]	<STPTx vs. STPTx vs. TPTx: 30 (22, 38) vs. 31 (24, 38) vs. 32 (22, 37)	<STPTx: 40% (67/167) STPTx: 57% (95/167) TPTx: 3% (7/167) Diagnosis of MEN1 before surgery: 36% vs. 82% vs. 80%, *p* < 0.0001	<STPTx vs. STPTx vs. TPTx Persistent PHPT: 25% vs. 3% vs. 0% Recurrent PHPT: 64% vs. 58% vs. 60% Persistent/recurrent PHPT: 84% vs. 61% vs. 60%, *p* = 0.0003 Second surgery: 69% 25% 20% Third surgery: 24% vs. 8% vs. 20% Fourth surgery: 1% vs. 2% vs. 0% Prolonged hypoparathyroidism: 9% vs. 7% vs. 40%, *p* > 0.05 Permanent laryngeal nerve palsy: 0%
[29]	PTx in 76.5% (13/17)	<STPTx: 38.5% (5/13) STPTx: 23.1% (3/13) STPTx and thymectomy: 15.4% (2/13) TPTx: 15.4% (2/13) <STPTx and hemithyroidectomy: 7.7% (1/13)	Persistent PHPT: 25% (3/17) Recurrent PHPT: 16.7% (2/13) Hypoparathyroidism: 41.7% (5/13)
[30]	NA	NA	N2: Reoperation: 25.9% (7/27) Recurrent PHPT: 71.4% (5/7) Persistent PHPT: 28.6% (2/7) Transient hypoparathyroidism: 66.7% (18/27) Permanent hypoparathyroidism: 14.8% (4/27) Transitory laryngeal nerve palsy: 11.1% (3/27) Permanent laryngeal nerve palsy: 3.7% (1/27)
[31]	NA	NA	N1: PTx in 80% (96/120) Persistent PHPT: 13.5% (13/96) Recurrent PHPT: 28.9% (24/83) Reoperation: 17.7% (17/96)
[32]	Median (Q1, Q3) age at PTx = 38 (22, 44) y	STPTx: 66.7% (20/30) TPTx: 33.3% (10/30)	Hypoparathyroidism: 23.33% (7/30)
[33]	Mean ± SD = 32 ± 12.7 y Age at most recent PTx: 42 ± 12 y	<STPTx: 42% (85/206) STPTx: 47% (95/204) TPTx and autotransplantation: 12% (24/206)	Prolonged hypoparathyroidism: 23% (47/206) Recovered hypoparathyroidism: 40% (19/47) At last follow-up: Aparathyroid: 1% (2/206) Hypoparathyroid: 13% (26/206) Euparathyroid: 54% (112/206) Hyperparathyroid: 31% (64/206) OR (95% CI) of prolonged hypoparathyroidism: Age at last operation: 1 (0.98, 1.03), *p* = 1 Female: 1.18 (0.61, 2.27), *p* = 0.6 4 or more glands resected: 6.02 (2.96, 12.24), *p* < 0.001 PTx before 2010: 2.07 (1.02, 4.23), *p* = 0.045 Immediate postoperative PTH < 15 ng/mL: 13.1 (3.61, 47.47), *p* < 0.001 OR (95% CI) of hypoparathyroidism recovery: Age at last operation: 0.96 (0.91, 1.01), *p* = 0.13 Female: 1.69 (0.47, 6.15), *p* = 0.42 4 or more glands resected: 0.19 (0.05, 0.72), *p* = 0.02 Reoperation: 1.02 (0.29, 3.6), *p* = 0.98
[34]	NA	PTx in 83.8% (57/68) <STPTx: 38.5% (22/57) STPTx: 61.5% (35/57)	Long-term remission: 56% (32/57) Persistent PHPT: 12.2% (7/57) Recurrent PHPT: 31.5% (18/57) Reoperation: 61% (11/18) Permanent hypoparathyroidism: 19.2% (11/57) Laryngeal nerve palsy: 0% Long-term remission and STPTx: OR (95% CI) = 1.7 (1.2–3.7, *p* < 0.001) Cinacalcet use: 33.8% (23/68)
[35]	N1 36.6 ± 14.3 y	N1 Did not undergo PTx: 21.1% (28/133) NCPHPT: 64.3% (18/28) PTx: 78.9% (105/133) <STPTx: 23.8% (25/105) STPTx: 39% (41/105) TPTx: 37.1% (39/105)	N1 Recurrent PHPT: 20% (21/105) Persistent PHPT: 11.4% (12/105) Permanent hypoparathyroidism: 12.4% (13/105) Reoperation: 14.3% (15/105)
[37]	43.4 ± 14.1 y	<STPTx: 36.35% (12/33) STPTx: 12.12% (4/33) TPTx: 51.51% (17/33)	N1 vs. N2 vs. N3: Persistent PHPT: 0% vs. 0% vs. 0% Recurrent PHPT: 25% (3/12) vs. 50% (2/4) vs. 5.9% (1/17), *p* = 0.076 Transient hypoparathyroidism: 0% vs. 0% vs. 23.5% (4/17), *p* = 0.154 Permanent hypoparathyroidism: 0% vs. 0% vs. 35.3% (6/17), *p* = 0.031 Parathyroid venous sampling vs. non-parathyroid venous sampling: Persistent PHPT: 0% vs. 0% Recurrent PHPT: 0% vs. 10% (1/10), *p* = 1.00 Transient hypoparathyroidism: 22.2% (2/9) vs. 10% (1/10), *p* = 0.582 Permanent hypoparathyroidism: 0% vs. 50% (5/10), *p* = 0.033 TPTx: 44.4% (4/9) vs. 100% (10/10), *p* = 0.011
[38]	NA	PTx in 68.18% (15/22) <STPTx: 20% (3/15)	Persistent PHPT: 6.7% (1/15) Recurrent PHPT: 0% Transient hypocalcaemia: 6.7% (1/15) Laryngeal nerve palsy: 0%
[39]	NA	<STPTx: 31.5% (28/89) STPTx: 25.8% (23/89) TPTx: 42.7% (38/89)	Persistent PHPT: 5.6% Recurrent PHPT: 36% Transient hypoparathyroidism: 49% Permanent hypoparathyroidism: 18% Permanent laryngeal nerve palsy: 0% Severe postoperative hypocalcaemia: 0% N1 vs. N2 vs. N3: Persistent PHPT: 14.2% vs. 0% vs. 2.6%, *p* = 0.052 Recurrent PHPT: 21.3% vs. 10.1% vs. 4.4%, N1 vs. N2 *p* = 0.03, N1 vs. N3 *p* = 0.001 Recurrence free survival: 101 (range 3301) vs. 139 (range 28–278) vs. 204 (range 75–396) months, N1 vs. N2 *p* = 0.018, N1 vs. N3 *p* = 0.049, N2 vs. N3 *p* = 0.35 Transient hypoparathyroidism: 0% vs. 26% vs. 100% Permanent hypoparathyroidism: 0% vs. 17% vs. 32%, N1 vs. N3 *p* = 0.01, N2 vs. N3 *p* = 0.06
[40]	NA	N: PTx: 73.2% (112/153)	NA

Abbreviations: <STPTx = less than subtotal parathyroidectomy; N = number of patients; NA = not available; OR = odds ratio; PTx = parathyroidectomy; SD = standard deviation; STPTx= subtotal parathyroidectomy; vs. = versus. Studies sub-groups have been introduced in Table 1; green font = post-surgery complications.

**Table 8:** Pre-operatory imaging evaluation of parathyroid tumours in MEN1 [36,38].

Reference	Preoperative Detection Rate and Key Findings in Pre-Surgery Imaging Scans
[36]	91.2% (51/56); N1 vs. N2: 87% vs. 93.9%, *p* = 0.33 **US features in N1 vs. N2:** Round lesions: 80% vs. 25.8%, *p* < 0.001 Irregular shape: 94% vs. 48.4%, *p* = 0.301 Vague boundary: 95% vs. 0%, *p* = 0.13 Heterogeneous: 96% vs. 45.2% *p* = 0.218 Abundant blood flow: 95% vs. 93.5%, *p* = 0.662
[38]	**US**: 91% (20/22) **SestaMIBI scintigraphy and SPECT/CT**: 96% (21/22) **FCH-PET/CT**: 96% (21/22) SUVmax adenoma vs. hyperplasia: 4.0 (range 1.8–13.4) vs. 3.9 (range 1.8–13.4), *p* = 0.14 Sensitivity: **US**: 60% **SestaMIBI and SPECT/CT**: 66% **US and sestaMIBI**: 76% **FCH-PET/CT**: 76% **US and FCH-PET/CT**: 84% **US and sestaMIBI and FCH-PET/CT**: 90% Specificity: **US**: 91% **SestaMIBI and SPECT/CT**: 87% **US and sestaMIBI**: 84% **FCH-PET/CT**: 92% **US and FCH-PET/CT**: 87% **US and sestaMIBI and FCH-PET/CT**: 81% Positive predictive value: **US**: 91% **SestaMIBI and SPECT/CT**: 83% **US and sestaMIBI**: 83% **FCH-PET/CT**: 91% **US and FCH-PET/CT**: 87% **US and sestaMIBI and FCH-PET/CT**: 83% Negative predictive value: **US**: 60% **SestaMIBI and SPECT/CT**: 71% **US and sestaMIBI**: 78% **FCH-PET/CT**: 79% **US and FCH-PET/CT**: 84% **US and sestaMIBI and FCH-PET/CT**: 88% Accuracy: **US**: 70% **SestaMIBI and SPECT/CT**: 76% **US and sestaMIBI**: 80% **FCH-PET/CT**: 84% **US and FCH-PET/CT**: 85% **US and sestaMIBI and FCH-PET/CT**: 85%

Abbreviations: FCH-PET/CT = fluorine-18 positron emission tomography associated with computed tomography; N = number of patients; sestaMIBI = methoxyisobutylisonitrile labelled with technetium-99 m; SPECT/CT= single-photon emission tomography associated with computed tomography; US = ultrasound; vs. = versus. Studies sub-groups have been introduced in Table 1; **bold font highlights type of imaging technique**.

**Table 9:** Pathological report in MEN1-associated parathyroid tumours [26,31,36,38,40].

Reference	Main Histological Findings
[26]	Hyperplasia: 59.4% (19/32) Adenoma: 9.4% (3/32) Carcinoma: 0%
[31]	N1 vs. N2 Multi-glandular involvement: 52.1% vs. 10%, *p* < 0.001 Carcinoma: 1% vs. 10%
[36]	N1 vs. N2: Multi-glandular involvement: 40% vs. 10%, *p* = 0.003 Mean parathyroid lesion numbers: 1.6 ± 0.91 vs. 1.1 ± 0.55, *p* = 0.002 Size: 1.68 ± 0.78 vs. 1.88 ± 0.73 cm, *p* = 0.349 Hyperplasia: 46.7% vs. 16.7%, *p* = 0.039
[38]	Adenomas: 26% Hyperplasia: 69% Thymus carcinoid tumours: 5%
[40]	Hyperplasia: 40.2% (45/112) Adenomas: 57.1% (64/112) Atypical parathyroid neoplasm: 1.8% (2/112) Parathyroid carcinoma: 0.9% (1/112)

Abbreviations: N = number of patients; vs. = versus. Studies sub-groups have been introduced in Table 1; histological report is provided according to terms designated by the original study.

**Table 10:** Quality of life analysis in parathyroidectomy candidates amid MEN1 confirmation [32].

Reference	Physical Component Summary	Mental Component Summary
[32]	Preoperative vs. 6 mo vs. 12 mo PCS: 76 (44–91) vs. 72 (51–92) vs. 80 (46–92), *p* = 0.71 PF: 88 (59–100) vs. 90 (64–100) vs. 85 (64–96), *p* = 0.57 RP: 100 (0–100) vs. 84 (0–100) vs. 100 (0–100), *p* = 0.22 BP: 88 (41–100) vs. 61 (41–100) vs. 72 (28–100), *p* = 0.23 GH: 62 (40–77) vs. 62 (44–82) vs. 60 (39–77), *p* = 0.55	Preoperative vs. 6 mo vs. 12 mo MCS: 66 (36–84) vs. 75 (33–87) vs. 76 (45–89), *p* = 0.23 VT: 60 (30–81) vs. 62 (40–80) vs. 65 (39–75), *p* = 0.51 SF: 69 (38–100) vs. 88 (57–100) vs. 82 (50–100), *p* = 0.04 RE: 100 (0–100) vs. 84 (0–100) vs. 100 (0–100), *p* = 0.22 MH: 66 (43–80) vs. 70 (44–88) vs. 72 (44–84), *p* = 0.23
	Symptomatic vs. asymptomatic PCS 61.2 (39.5–83.0) vs. 92.5 (83.5–94.2), *p* = 0.0051 PF 80.0 (40.0–90.0) vs. 100.0 (90.0–100.0), *p* = 0.0093 RP 50.0 (0.0–100.0) vs. 100.0 (100.0–100.0), *p* = 0.08 BP 62.0 (30.0–100.0) vs. 94.0 (62.0–100.0), *p* = 0.14 GH 47.0 (37.0–67.0) vs. 77.0 (62.0–82.0), *p* = 0.0062	Symptomatic vs. asymptomatic MCS 56.0 (28.5–75.5) vs. 82.0 (46.2–92.2), *p* = 0.04 VT 45.0 (25.0–70.0) vs. 80.0 (55.0–90.0), *p* = 0.01 SF 50.0 (25.0–100.0) vs. 88.0 (38.0–100.0), *p* = 0.21 RE 100.0 (0.0–100.0) vs. 100.0 (33.0–100.0), *p* = 0.30 MH 56.0 (28.0–72.0) vs. 76.0 (64.0–84.0), *p* = 0.02
	Pain score and PCS: r = −0.60, CI = (−0.78, −0.26), *p* = 0.0009	MCS and remnant parathyroid volume at 6 mo: r = 0.3807, *p* = 0.038
	Total parathyroid volume and RP: r = −0.44, CI = (−0.70, −0.09), *p* = 0.01)	Postoperative MCS: 80.25 (54.88–92.63) vs. 32.25 (16.38–83.75), *p* = 0.0365)
	PCS and remanent parathyroid volume at 12 mo: r = 0.3625, *p* = 0.049	
	1–2 comorbidities vs. 3–4 comorbidities: Preoperative PCS: 88.0 (65.38–93.63) vs. 39.50 (30–75–63–38), *p* = 0.0015 Postoperative PCS: 84.5 (63.38–84.50) vs. 28.75 (20.25–74.13), *p* = 0.0031	

Abbreviations: BP = bodily pain; GH = general health; MCS = mental component summary; MH = mental health; mo = months; PCS = physical component summary; PF = physical functioning; RE = role-functioning emotional; RP = role-functioning physical; SF = social functioning; vs. = versus; VT = vitality. Studies sub-groups have been introduced in Table 1.

## Abbreviations

The following abbreviations are used in this manuscript:

APN	atypical parathyroid neoplasm
BMD	bone mineral density
CI	confidence interval
DXA	Dual-Energy X-Ray Absorptiometry
F	females
F:M	female-to-male ratio
FCH-PET/CT	fluorine-18 positron emission tomography/computed tomography
GEP-NETs	gastro-entero-pancreatic neuroendocrine tumours
HR-pQCT	high-resolution peripheral quantitative computed tomography
IQR	interquartile range
MEN	multiple endocrine neoplasia
M	males
MPHPT	MEN1-related primary hyperparathyroidism
N	number of patients
n	number of studies
NET	neuroendocrine tumours
NA	not available
OR	odds ratio
PHPT	primary hyperparathyroidism
PTH	parathyroid hormone
PC	parathyroid carcinoma
PTx	parathyroidectomy
PitNETs	pituitary neuroendocrine tumours
STPTx	subtotal parathyroidectomy
<STPTx	less than subtotal PTx
SD	standard deviation
sestaMIBI	methoxyisobutylisonitrile labelled with technetium-99 m
SGE	single gland excisions
TPTx	total parathyroidectomy
TGF	tumour growth factor
TBS	trabecular bone score
vs.	versus
